# Immunological biomarkers and gene signatures predictive of radiotherapy resistance in non-small cell lung cancer

**DOI:** 10.3389/fimmu.2025.1574113

**Published:** 2025-05-29

**Authors:** Jie Lv, Chun-yang Yu, Yao-zu Xiong, Ting-ting Dai, Xiao-chu Hu, Peng Pan, Shun Yue, Chang-hua Yu

**Affiliations:** ^1^ Department of Radiotherapy, The Affiliated Huaian No. 1 People’s Hospital of Nanjing Medical University, Huai’an, China; ^2^ Department of Cardiology, The Affiliated Huaian No. 1 People’s Hospital of Nanjing Medical University, Huai’an, China; ^3^ Department of Oncology, The Affiliated Huaian No. 1 People’s Hospital of Nanjing Medical University, Huai’an, China

**Keywords:** NSCLC, radiotherapy resistance, bioinformatic analysis, bioinformatics analysis, immunological biomarkers

## Abstract

**Introduction:**

A significant challenge in treating non-small cell lung cancer (NSCLC) is its inherent resistance to radiation therapy, leading to poor patient prognosis. This study aimed to identify key genes influencing radiotherapy resistance in NSCLC through comprehensive bioinformatics analysis.

**Methods:**

A total of 103 common genes were identified, enriched in critical biological pathways such as coagulation, complement activation, growth factor activity, and cytokine signaling. Using advanced machine learning techniques like SVM-RFE, LASSO regression, and random forest algorithms, four pivotal genes—TGFBI, FAS, PTK6, and FA2H—were identified.

**Results:**

TGFBI showed the strongest correlation with NSCLC prognosis as indicated by a diagnostic nomogram. Additionally, significant differences in immune cell infiltration, particularly involving naive B cells and M0 macrophages, were noted between high-risk and low-risk patients.

**Discussion:**

The study suggests that targeting pathways regulating macrophage polarization or enhancing naive B cell activation could play a crucial role in addressing radiotherapy resistance. The findings highlight the potential therapeutic targets, thereby advancing the understanding of the molecular mechanisms underlying radiotherapy resistance in NSCLC, with implications for improving patient management and outcomes.

## Highlights

- Identified 103 genes enriched in NSCLC radiotherapy resistance pathways. Using machine learning, pinpointed four key genes (TGFBI, FAS, PTK6, FA2H), with TGFBI highly correlated with prognosis.

- Found significant immune cell infiltration differences between high- and low-risk NSCLC patients, hinting at new therapeutic strategies involving macrophage polarization and B cell activation.

- Developed a prognostic model with high diagnostic accuracy based on identified genes, advancing understanding of radiotherapy resistance mechanisms and potentially improving patient outcomes.

## Introduction

NSCLC is still the most common cause of cancer-related mortality globally, accounting for around 85% of all occurrences of lung cancer (1).

Lung cancer caused over 1.8 million deaths worldwide in 2020, highlighting its aggressive nature and treatment challenges. Despite advancements in surgery, chemotherapy, and targeted therapies (2), the five-year survival rate for NSCLC patients remains low. This is mostly because to late-stage diagnosis and the emergence of treatment resistance (3). Current diagnostic methods, including imaging and histopathological examination, often fail to detect early-stage disease, limiting the effectiveness of potentially curative interventions (4).

The current standard treatment strategies mainly include: for early-stage NSCLC patients, surgery is the preferred treatment approach (5), while for patients with locally advanced or inoperable disease, radiation therapy and concurrent or sequential chemotherapy are the conventional regimens (6–8). In recent years, significant progress has been made in molecular targeted therapy, with targeted inhibitors for driver gene mutations such as EGFR, ALK, and ROS1 (e.g., EGFR-TKI, ALK-TKI) becoming standard treatment options for advanced NSCLC (9, 10). Additionally, immune checkpoint inhibitors (e.g., PD-1/PD-L1 inhibitors), either alone or in combination, have also demonstrated good efficacy in advanced NSCLC without driver gene mutations (11). However, despite the improvements in survival for patients, the emergence of resistance and the variability in treatment response among individuals remain major clinical challenges (12, 13).

Radiotherapy is essential for managing NSCLC, especially in patients with inoperable tumors or those unable to undergo surgery (14). However, its effectiveness is often reduced by radioresistance, where cancer cells adapt to resist radiation’s cytotoxic effects (15). This resistance involves complex molecular mechanisms, including changes in DNA repair, cell cycle regulation, apoptosis, and the tumor microenvironment (6). Identifying genetic factors of radioresistance is vital for improving therapeutic responses and patient outcomes. Research indicates that specific genes and signaling pathways are crucial in radioresistance across various cancers. For example, increased epidermal growth factor receptor (EGFR) expression is associated with radioresistance in head and neck squamous cell carcinoma, suggesting that EGFR inhibition could enhance radiation therapy effectiveness (16). Similarly, radioresistance in breast cancer is linked to alterations in the PI3K/AKT pathway, with inhibitors being investigated as potential radiosensitizers (17). These findings highlight the benefits of targeting molecular pathways to overcome radioresistance and improve radiotherapy efficacy in NSCLC.

Our methodology combined Support vector machine-recursive feature elimination(SVM-RFE), Gene set variation analysis(LASSO) regression, and random forest algorithms to identify key genes associated with radioresistance. We developed diagnostic nomograms and predictive models to assess the clinical relevance of these genes. By elucidating the genetic basis of radioresistance, we aim to advance targeted treatment strategies, potentially improving radiotherapy efficacy and patient survival.

## Materials and methods

### Data sources

This study utilized publicly accessible, cost-free data from the Gene Expression Omnibus (GEO) and The Cancer Genome Atlas Program (TCGA). Whole genome expression profiles for NSCLC were retrieved from these databases using the R packages ‘GEOquery’ and ‘TCGAbiolinks.’ The dataset GSE197236,derived from the GPL26963 Agilent-085982 Arraystar human lncRNA V5 microarray, includes three control samples and three radiation-resistant samples from A549 lung adenocarcinoma cell lines. Additionally, the dataset GSE253564, using the Illumina NovaSeq 6000 platform (GPL24676 for Homo sapiens), comprises 32 samples. This study specifically examines the Arm1 group, with 10 lung adenocarcinoma samples treated with PD-L1, and the Arm2 group, with 10 samples receiving a combination of PD-L1 and radiotherapy. The datasets GSE197236 and GSE253564 were used to identify genes linked to radiotherapy resistance in NSCLC. The TCGA-LUAD dataset consists of 600 samples, including 539 from primary tumors, 2 from recurrent tumors, and 59 from nearby normal tissues. However, the two recurrent tumor samples were not included in this analysis. The TCGA-LUAD dataset was utilized for patient classification and prognostic assessment based on genes associated with radiotherapy resistance. The ComBat function from the R package ‘sva’ was applied to correct dose effects caused by non-biotechnological biases (18). The adjustment effect was managed using principal component analysis (PCA).In this study, we followed the data access policies of each database and assessed the impact of rectification using PCA.

### Raw data processing

GSE253564 provides normalized data (FPKM) on GEO, making it unsuitable for differential analysis.This research obtained raw data from the SRA database and aligned the sequencing information to the GRCh38 reference genome using the STAR alignment tool. (v2.7.11b), obtaining gene expression data tables using featureCount and the ENSEMBL Homo_sapiens.GRCh38.112.chr.gtf annotation file.

### Differential expression analysis

This study utilized the limma package to analyze GSE197236 chip data, identifying differentially expressed genes based on criteria of p<0.05 and |log2 Fold Change|>1.GSE253564 is second-generation sequencing data, and this study used DESeq2 for differential analysis, with the same criteria for identifying differentially expressed genes.

### GO/KEGG enrichment analysis

The Gene Ontology (GO) enrichment analysis encompasses three categories: biological process (BP), molecular function (MF), and cellular component (CC) (19). Metabolic pathways were identified using the Kyoto Encyclopedia of Genes and Genomes (KEGG) (20). The R package ‘clusterProfiler’ (version 4.2.2) was employed for both GO and KEGG analyses, with significance set at p< 0.05 (21).

### GSEA

Gene Set Enrichment Analysis (GSEA) is a widely utilized computational technique in bioinformatics for uncovering detailed insights in genomic expression data (22). To delve deeper into how key genes influence NSCLC, we conducted a single-gene GSEA analysis. We computed log2Fold Change values for other genes relative to the expression levels of key genes. Using the R package ‘clusterProfiler’ (version 4.2.2), we ranked all genes by their log2Fold Change values and performed 1,000 permutation analyses on gene sets. The c2.cp.kegg.v7.5.1.symbols collection from the Molecular Signatures Database (MSigDB) was used as the reference gene set (22–24). Gene sets with an adjusted p-value<0.05 were deemed significantly enriched.

### GSVA

Gene Set Variation Analysis (GSVA) is an unsupervised, non-parametric approach for evaluating gene set enrichment, facilitating the investigation of links between biological pathways and gene features from expression data. To examine functional differences between high-risk and low-risk groups, we used the ‘c2.cp.kegg.v7.5.1.symbols’ gene set from the MSigDB database (http://software.broadinstitute.org/gsea/msigdb) as a reference. GSVA was performed using the R package ‘GSVA’ (version 1.42.0), and results were visualized with the R package ‘pheatmap’ (version 1.0.12). We utilized 50 hallmark gene sets from the MSigDB database as reference points. The Sample Gene Set Enrichment Analysis(ssGSEA) function from the GSVA package calculated GSVA scores for each gene set across various samples. The limma software analyzed differences in GSVA scores between high-risk and low-risk groups.

### Construction of diagnostic nomogram

A nomogram model for diagnosing non-small cell lung cancer was developed using the R package rms, incorporating risk scores derived from key gene expression levels and pertinent clinical data. The cumulative risk score aggregates individual gene risk scores to offer a comprehensive risk assessment. The nomogram’s diagnostic effectiveness for NSCLC was assessed through calibration.

### Receiver operating characteristic curve

The receiver operating characteristic (ROC) curve is an essential tool for assessing diagnostic test performance, illustrating the relationship between sensitivity and specificity as continuous variables. The area under the curve (AUC), derived from sensitivity and specificity measures, is a widely used parameter in graphical analysis. In this case, the R package “pROC” was used to construct ROC curves, allowing the estimation of the AUC for various screening feature genes and assessing their diagnostic importance (25). Typically, AUC values vary between 0.5 and 1, with an AUC value approaching 1 signifying outstanding diagnostic accuracy.

### Screening key genes

SVM-RFE is a sophisticated machine learning technique focused on training feature subsets from different categories to enhance the feature set and pinpoint the most predictive variables. The “glmnet” package in R was employed for LASSO regression, facilitating the computation and selection of linear models while retaining significant variables. For LASSO classification, we utilized binomial distribution variables, where the model was developed by choosing the minimum error, despite using only 10 cross-validation variables.

The “RandomForest” function was employed for random forest analysis, with the mtry parameter set to minimize error and consistent image values chosen for the ntree parameter. The analysis identified the top 50 crucial genes based on mean decrease accuracy (MDA) and mean decrease Gini (MDG) of feature weights. Key genes were determined by cross-referencing those identified through LASSO regression, SVM-RFE, and random forest methods.

### Building the ceRNA network

We utilized miRTarBase, starbase2.0, and miRDB databases to perform reverse prediction of microRNAs, identifying long non-coding RNAs (lncRNAs) that share microRNAs with key genes, thereby aiding in the construction of the ceRNA network.

### RBP-mRNA regulatory network

This research employed the StarBasehttps: We utilized the starbase.sysu.edu.cn platform to explore non-coding RNA (ncRNA) relationships by analyzing CLIP-seq, degradome-seq, and RNA-RNA interaction data. This analysis focused on the expression levels of mRNA and RNA-binding proteins (RBPs).In a disease context, we applied specific thresholds (p<0.05, lusterNum≥5, clipExpNum≥5) to identify significant mRNA-RBP pairs. Subsequently, we constructed an RBP-mRNA network using Cytoscape to visualize and further investigate these interactions.

### TF-mRNA regulatory network

The hTFtarget database provides an extensive collection of transcription factor(TF) target regulations, compiles from a wide range of human TF ChIP-Seq data, which includes 7,190 experimental samples from 659 transcription factors (http://bioinfo.life.hust.edu.cn/hTFtarget#!).This data encompasses 569 conditions, including 399 cell lines,129 tissue or cell types, and 141 treatment methods.

### Building prognostic models

To evaluate the prognostic importance of phenotype-related differentially expressed genes(DEGs) on overall survival (OS) within tumor cohort. Genes with p<0.05 were considered to be highly associated with OS and their selection for further research. We randomly assigned tumor samples with clinical information to two sets in a 7:3 ratio:A training set of 279 samples and a validation set of 141 samples were utilized. The LASSO Cox regression model, implemented via the ‘glmnet’ R package, was employed to refine candidate genes and develop the prognostic model (26). The penalty parameter (λ) was determined using the minimal criterion, and the risk score was calculated using the derived equation.


$$riskScore=\sum_{i=1}^{n}Coef\left(gene_{i}\right)*Expression\left(gene_{i}\right)$$

$$Coef\left(gene_{i}\right),\;Expression\left(gene_{i}\right)$$

The training set samples were divided into low-risk and high-risk groups based on the average risk score. The prognostic significance was evaluated using Kaplan-Meier survival curves and statistical significance was determined with a log-rank test. The prognostic model’s performance was assessed via the ROC curve, with AUC values ranging from 0.5 to 1, where values closer to 1 indicate greater accuracy. The validation set underwent the same processing to confirm the model’s predictive effectiveness.

### Immune infiltration analysis

Sample Gene Set Enrichment Analysis (ssGSEA) extends Gene Set Enrichment Analysis (GSEA) by computing an enrichment score for each sample and gene set (27). This score reflects the level of coordinated expression changes, either upregulation or downregulation, within a gene set for a specific sample. Unlike traditional GSEA, which evaluates enrichment scores for groups of samples and gene sets, ssGSEA provides scores for individual sample and gene set pairs. This study utilized immune cell marker gene expression data sourced from the Tumor Immune System Interaction Database (TISIDB) (28).

The dataset encompasses a range of immune cell types, including various subtypes of CD8 and CD4 T cells, T helper cells, B cells, natural killer cells, dendritic cells, and other immune cells such as macrophages, eosinophils, mast cells, monocytes, and neutrophils. We analyzed gene expression profiles to determine the relative enrichment scores for these immune cell types. Using the R package ‘ggplot2’,we visualized differences in immune cell infiltration between NSCLC samples categorized into high and low groups (29). Additionally, rank-sum tests were performed to compare mutation frequencies between high-risk and low-risk groups, with results visualized using the maftools package.

### Somatic mutation analysis

Utilizing the “maftools” R package, we examined mutation data to compare tumor mutation burden between high-risk and low-risk groups (30), starting with the calculation of total mutation counts across samples. Next, we identified genes with mutation counts greater than 40.To compare the mutation frequencies we applied rank-sum tests and the maftools package to visualize the data.

### Statistical analysis

The study utilized R software v4.1.2 for statistical analysis. Spearman correlation tests assessed relationships between two variables. The Wilcoxon test compared differences between two groups, and the Kruskal-Wallis test evaluated variations across three or more groups. Statistical significance was determined by a two-sided p-value< 0.05.

## Results

**Figure 1:**
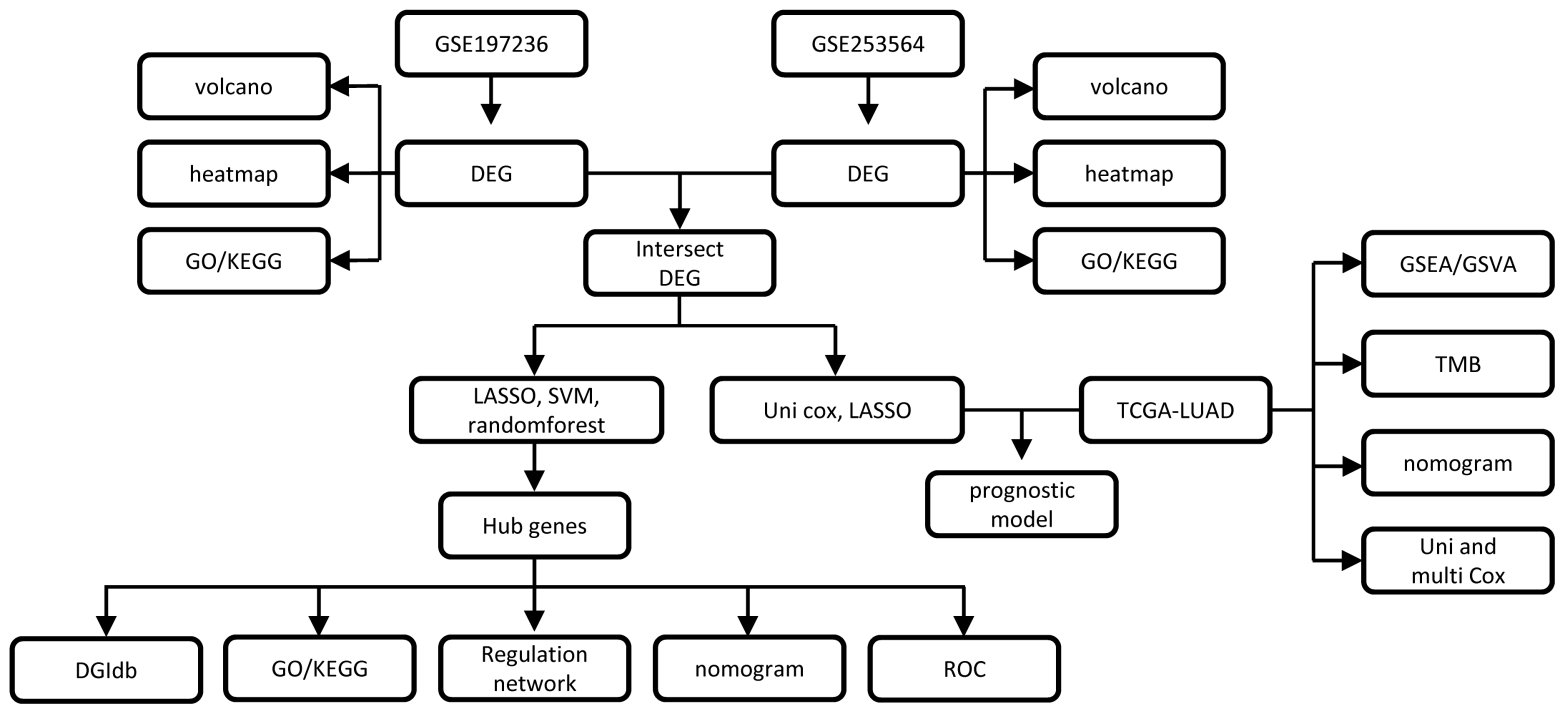
Analysis flowchart.

The analysis flowchart of this study is shown in **Figure 1**.

### Screening for radiotherapy tolerance genes

**Figure 2:**
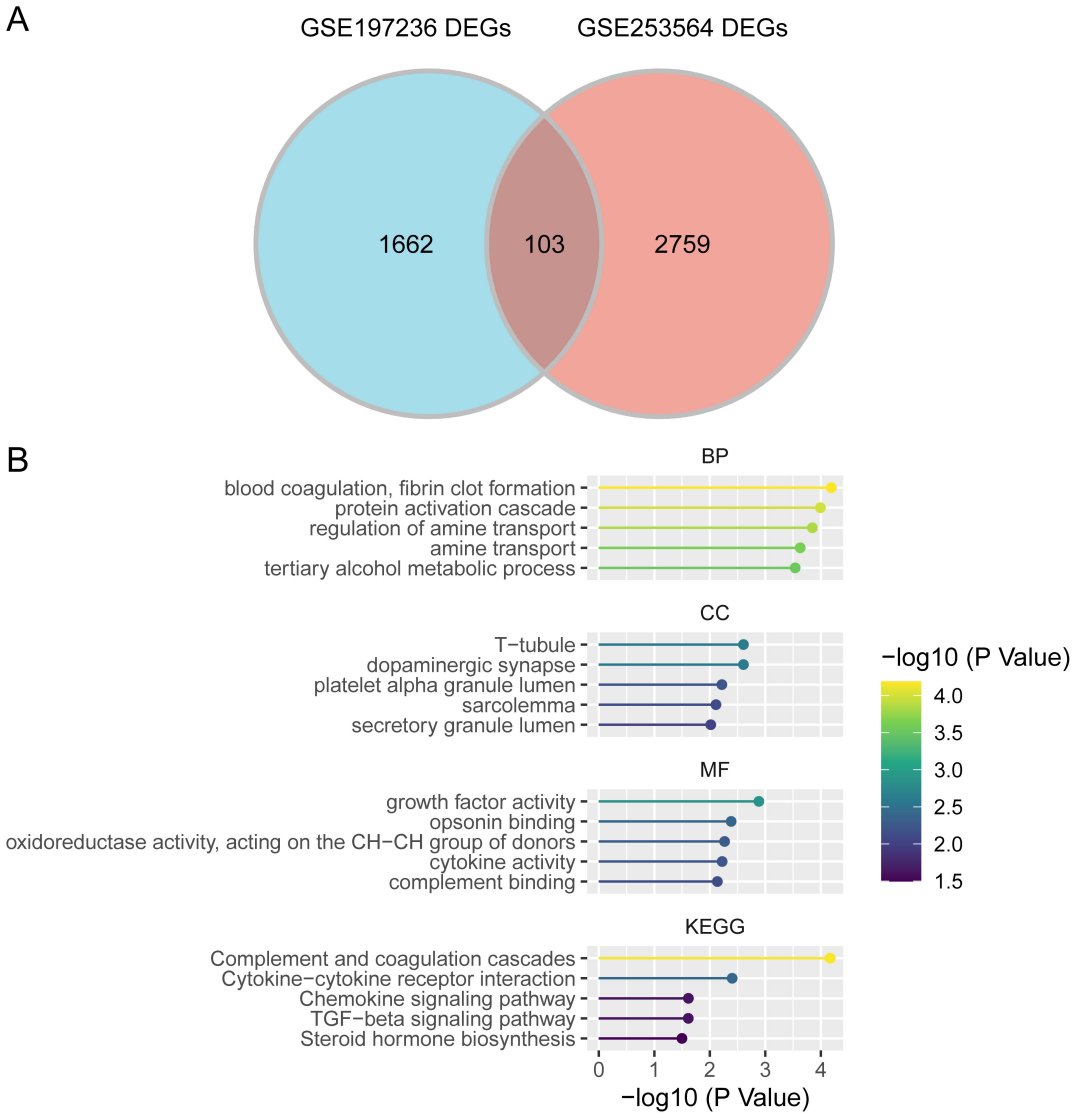
Screening of radiotherapy tolerance-related genes. **(A)**. Intersection of differentially expressed genes from two datasets. **(B)**. GO/KEGG enrichment analysis.

To identify genes associated with radiotherapy tolerance, we conducted a differential analysis using GSE197236 (radiation v.s. control) (**Supplementary Figures 1A, B** and **Supplementary Table S1**), using GSE253564 (anti PD-L1 + radiation therapy v.s. anti PD-L1 therapy).Using a threshold of p<0.05 and |log2 Fold Change| ≥ 1, we identified 2,188 DEGs, comprising 112 upregulated and 2,076 downregulated genes. To illustrate our findings, we generated a volcano plot (**Supplementary Figure 2A**) and a heatmap showcasing the top 20 DEGs between the two groups (**Supplementary Figure 2B**). We subsequently identified the overlap between the two sets of DEGs mentioned earlier, resulting in a total of 103 genes that are associated with radiotherapy resistance in NSCLC (**Figure 2A**; **Supplementary Table S3**). The enrichment analysis of the selected differential genes showed significant associations with pathways involved in DNA damage, cytokines, and p53 (**Supplementary Figure 1C**). GO (**Supplementary Table S4**) and KEGG enrichment analyses (**Supplementary Table S5**) demonstrated a notable enrichment of gene sets associated with the process of blood coagulation. The processes of fibrin clot formation, complement activation, and the activities of growth factors and cytokines(including cytokine-cytokine receptor interaction and chemokine signaling) suggest potential roles in mediating radiotherapy resistance (**Figure 2B**).

### Machine learning algorithms for selecting key genes

We employed three machine learning algorithms:LASSO, random forest, and SVM-RFE to screen the intersection of 103 genes (**Supplementary Figure 3**), ultimately identifying a total of 4 key genes (**Supplementary Table S4**). The enrichment analysis of the four key genes indicated significant enrichment in gene sets associated with tissue development, cellular component organization or biogenesis, positive regulation of protein phosphorylation and phosphorus metabolic processes, nuclear body, organelle subcompartment, trans-Golgi network, ruffle, Golgi apparatus subcompartment,identical protein binding, integrin binding, extracellular matrix structural constituent, kinase binding, transmembrane signaling receptor activity, Platinum drug resistance, p53 signaling pathway, Necroptosis, MAPK signaling pathway, and Apoptosis (**Supplementary Figure 3F**).

### Constructing a diagnostic nomogram based on key genes

**Figure 3:**
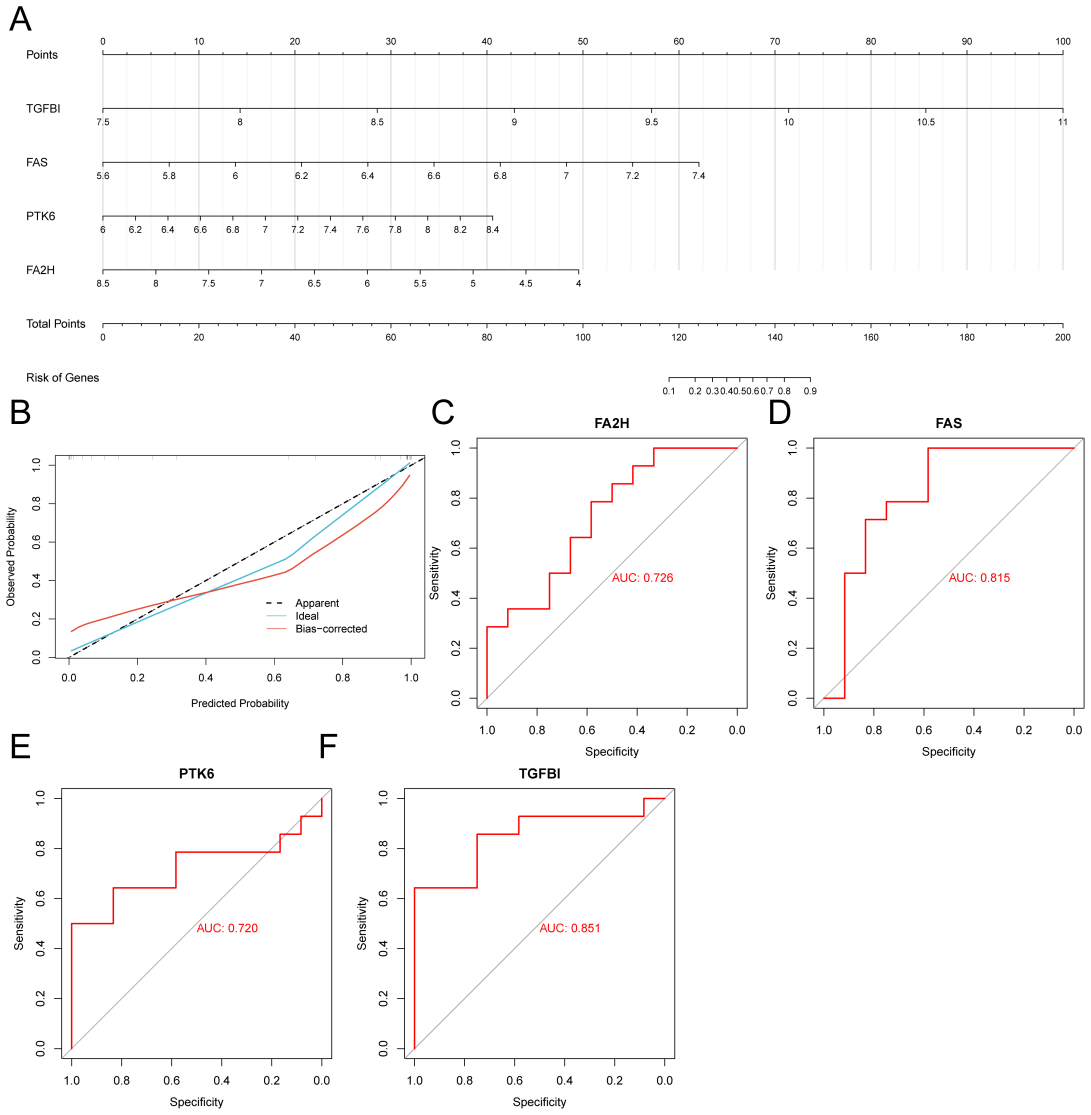
Construction of diagnostic nomograms. **(A)** Key gene diagnostic nomogram. **(B)** Calibration curve of the diagnostic nomogram. **(C)** ROC curve of FA2H. **(D)** ROC curve of FAS. **(E)** ROC curve of PTK6. **(F)** ROC curve of TGFB1.

Based on key genes, we developed a diagnostic nomogram, as illustrated in **Figure 3A**. This nomogram indicates that transforming growth factor beta-induced(TGFBI) exhibits the strongest correlation with the prognosis of NSCLC, followed closely by Fatty acid synthase(FAS).To assess the accuracy of our diagnostic nomogram, we utilized calibration curves, shown in **Figure 3B**. The results demonstrated that the predicted values from the nomogram closely aligned with the actual observed values, indicating a robust predictive capability. Additionally, we conducted ROC analysis on the key genes, revealing that all four key genes displayed strong classification ability, with an AUC>0.7, as presented in **Figures 3C–F**.

### Constructing the regulatory network of key genes

To investigate the molecular mechanisms underlying key genes in NSCLC we created an mRNA-miRNA-lncRNA regulation network (**Supplementary Figure 4A** and **Supplementary Table S7**). Using the Starbase database, we screened for miRNAs and lncRNAs that regulate FAS,TGFB1, and Fatty acid 2-hydroxylation (FA2H).

The StarBase database was employed to identify and obtain mRNA/RBP (RNA binding protein) pairs of four key genes. An RBP-mRNA network (**Supplementary Figure 5A**, **Supplementary Table S9**) was built from the interactions among target genes provided by the online dataset. To investigate the transcriptional regulatory factors affecting the key genes, we constructed a regulatory factor network using the TRRUST database (**Supplementary Figure 5B**, **Supplementary Table S8**). The results showed that FAS is regulated the most, and SP1 can simultaneously regulate both FAS and PTK6.

We used DGIdb to screen potential drugs that regulate key gene activities (**Supplementary Figure 6** and **Supplementary Table S10**). The results showed that EDELFOSINE, VB−111,IUPHAR.LIGAND:2011,IUPHAR.LIGAND:2025,and IUPHAR.LIGAND:2006 are potential target drugs for FAS.

### Building a prognostic model

**Figure 4:**
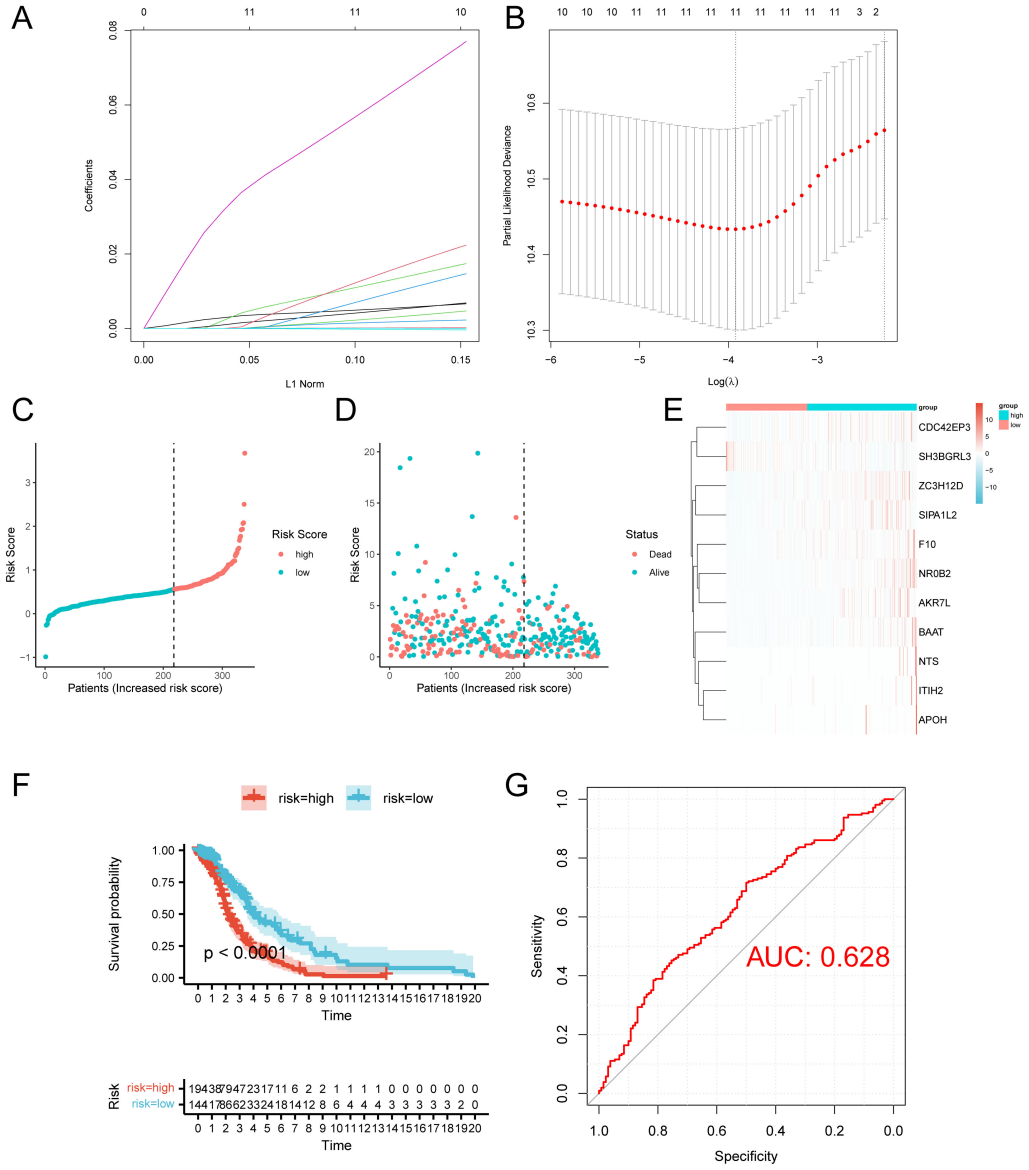
Construction of the prognostic model. **(A)** Variable path diagram of LASSO regression. **(B)** LASSO regression cross-validation graph. **(C)** Training set risk curve. **(D)** Training set survival status. **(E)** Heatmap of prognostic-related gene expression levels of the training group. **(F)** Survival analysis of the test group. **(G)** ROC curve of the training group.

Utilizing the intersecting genes and clinical data obtained from TCGA-LUAD, we initially divided the samples into training and testing groups at 7:3 ratio. We employed univariate Cox regression analysis to identify prognostic-related genes from the intersecting genes (**Supplementary Table S11**) and further refined this selection using LASSO regression (**Figures 4A, B**; **Supplementary Table S12**).We assessed ach individual sample’s risk values and applied the surv_cutpoint method to find the threshold that distinguishes high-risk from low-risk groups. This threshold categorized samples into high- and low-risk groups. Survival analysis indicated a notable disparity in survival outcomes between the high-risk and low-risk groups, demonstrating that individuals in the high-risk group experienced significantly shorter survival times compared to those in the low-risk group (**Figures 4C–F**). We proceeded to plot the ROC curves for both groups (**Figure 4G**), which indicated that our model demonstrated good classification ability for the data, with an AUC greater than 0.6, suggesting a certain predictive value for patient prognosis. Survival analysis indicated notable differences in outcomes between high-and low-risk groups, with the high-risk group exhibiting a significantly shorter median survival time.(Refer to **Figures 4E, F**).

Additionally, we generated the risk curves, survival status, and created heatmaps illustrating the expression levels (**Supplementary Figures 7A–F**). We produced a risk curve, assessed survival status, and generated a heatmap to illustrate the expression levels of prognosis-related genes in high- and low-risk group.

### Immune infiltration analysis

**Figure 5:**
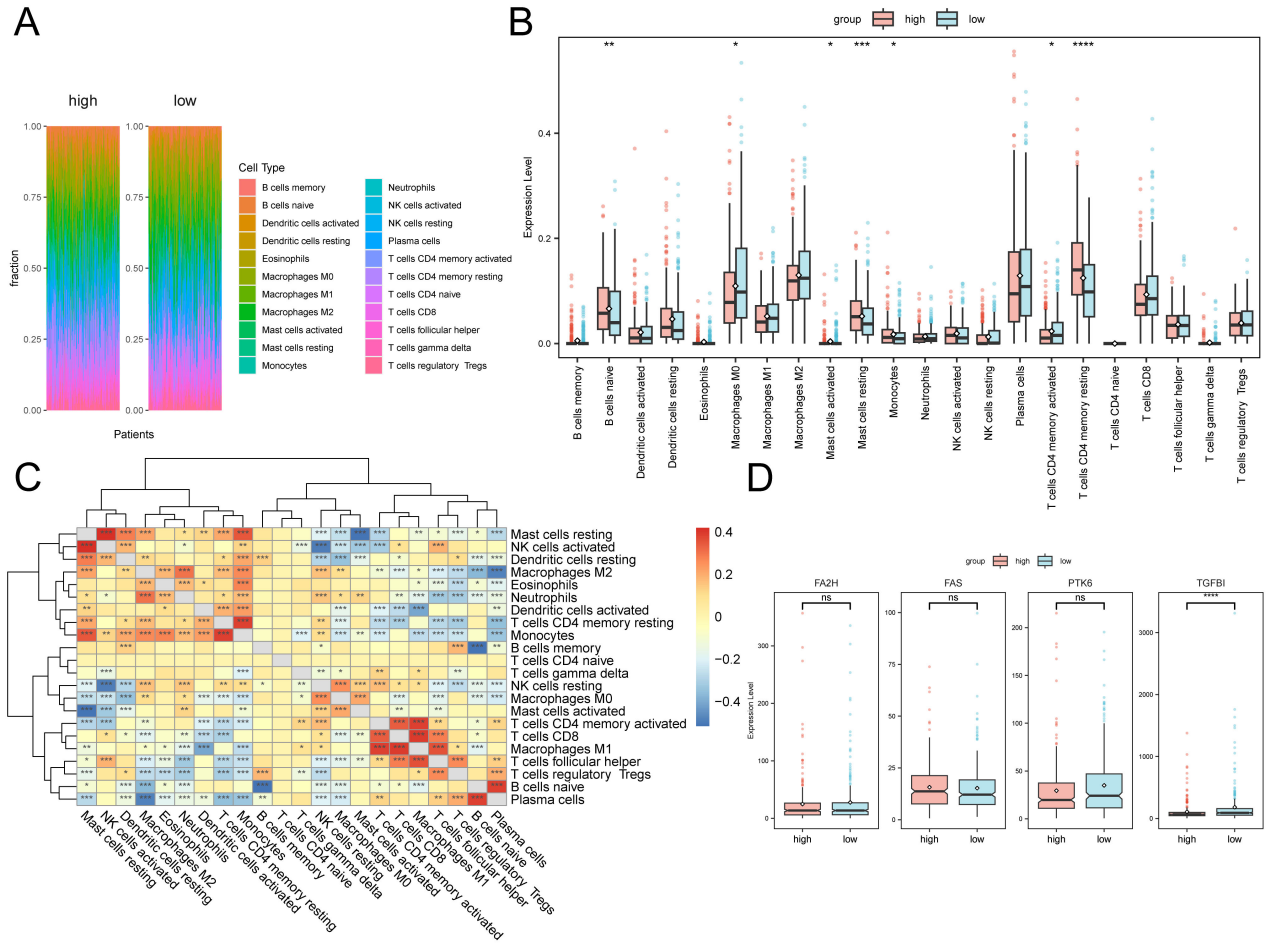
Immune infiltration analysis. **(A)** Proportion of immune cells in different samples. **(B)** Box plot of immune cell infiltration levels between high and low-risk group. **(C)** Box plot of immune cell infiltration levels. **(D)** Box plot of the expression levels of prognostic-related genes in high and low-risk group. The asterisk represents the P value: ****p<0.0001, ***p<0.001, **p<0.01, *p<0.05. ns, no significance.

To investigate the role of immune cells in tumor development, we analyzed the infiltration levels of 28 immune cell types in high- and low-risk groups. The analysis revealed significant differences in naive B cells, M0 macrophages, activated and quiescent mast cells, monocytes, and both activated and dormant CD4+ memory T cells (**Figures 5A, B**). The correlation analysis identified a statistically significant positive association between naive B cells and plasma cells, resting mast cells and activated NK cells, and resting CD4 memory T cells and monocytes (**Figure 5C**). Conversely, a significant negative relationship was observed between M2 macrophages and plasma cells infiltration levels, as well as between activated dendritic cells and M1 macrophages. (**Figure 5C**). Analysis of gene expression levels related to prognosis (**Figure 5D**) revealed that only TGFBI showed a statistically significant difference, with higher expression in the low-risk group.

We conducted a correlation analysis between the expression levels of prognosis-related genes and immune cell infiltration. Since only T cells CD4 memory resting, Mast cells resting, B cells naïve, Macrophage M0, Mast cells activated, and Monocytes indicated notable variations in two groups, we showed the top 6 strongest correlations genes with prognosis-related genes in the scatter plots. The infiltration levels of FAS was highly correlated with T cells CD4 memory resting, Monocytes, and Mast cells resting, and showed a negative correlation with B cells naïve (**Supplementary Figures 8A, C ,D, F**).Furthermore, there existed a negative correlation between the infiltration level of naïve B lymphocytes and TGFBI, whereas a positive connection with FA2H (**Supplementary Figures 8B, E**).

### GSEA and GSVA

GSEA and GSVA were utilized to examine expression differences in various pathways between high and low-risk groups. The GSEA results revealed that the low-risk group had considerably higher levels in the Pentose Phosphate and Proteasome Pathway (**Supplementary Figures 9A, B** and **Supplementary Table S13**). In contrast, the high-risk group showed significant enrichment in the Phosphatidylinositol signaling system and vascular smooth muscle contraction (**Supplementary Figures 9C, D** and **Supplementary Table S13**).

GSVA analysis revealed significant enrichment of the KEGG_GNRH_SIGNALING_PATHWAY, KEGG_ALDOSTERONE_REGULATED_SODIUM_REABSORPTION, and KEGG_VASCULAR_SMOOTH_MUSCLE_CONTRACTION pathways in the high-risk group.

**Figure 6:**
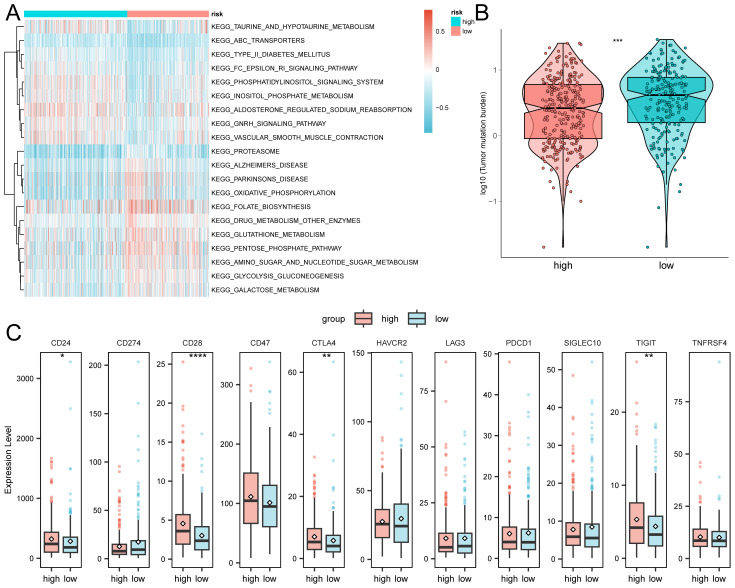
GSVA analysis of high and low-risk groups. **(A)** The top 20 pathways. **(B)** Analysis of tumor mutation burden. **(C)** Expression levels of immune checkpoints. The asterisk represents the P value: ****p<0.0001, ***p<0.001, **p<0.01, *p<0.05.

The pathways KEGG_FOLATE_BIOSYNTHESIS, KEGG_DRUG_METABOLISM_OTHER_ENZYMES,and KEGG_GLUTATHIONE_METABOLISM were significantly enriched in the low-risk group (**Figure 6A**; **Supplementary Table S14**). Furthermore, mutation burden analysis revealed a higher mutation load in the low-risk group compared to the high-risk group (**Figure 6B**). Immune checkpoint analysis showed significant overexpression of CD24, CD28, CTLA4, and TIGIT in the high-risk group (**Figure 6C**).

### Diagnostic nomogram

Utilizing univariate and multivariate Cox regression models, we examined the relationship between survival outcomes and clinical data. The results revealed significant associations of Risk, Stage N, and Oncogene activity with survival status. We then developed a diagnostic nomogram incorporating these three clinical variables and validated it using ROC curves. The findings demonstrated that the nomogram possessed predictive capability (AUC > 0.6) (**Supplementary Figure 10**).

## Discussion

NSCLC is the most prevalent and deadly form of lung cancer (2), posing a significant public health threat due to its high mortality rate and poor prognosis at advanced stages (4, 31). Recent progress in molecular biology and genetics has identified key genetic mutations and pathways critical to NSCLC development, enhancing our understanding of its causes (17, 32). These discoveries have facilitated targeted therapies and personalized treatments, which show promise in improving survival rates and reducing disease progression (6). Moreover, combining radiotherapy and immunotherapy with conventional treatments has shown potential in overcoming therapeutic resistance and enhancing NSCLC management efficacy (33). This study seeks to identify genes involved in radiation therapy resistance by analyzing radiation-resistant related genes in NSCLC. Through this research, we hope to provide new insights into the mechanisms of radiotherapy resistance in NSCLC and to identify potential biomarkers or therapeutic targets for overcoming radiotherapy resistance in clinical practice.

Through differential analysis of the GSE197236 and GSE253564 datasets, We managed to identify 103 genes linked to radiotherapy tolerance in NSCLC, marking a significant achievement in our screening process. Our analysis of these genes revealed important connections to DNA damage response mechanisms, cytokine signaling, and pathways related to the p53 tumor suppressor protein. The discovery indicates that these pathways could be of pivotal importance in determining the level of tolerance to radiotherapy. The role of DNA damage repair mechanisms in enabling radiotherapy tolerance has been thoroughly investigated. Radiotherapy works by inducing DNA double-strand breaks in cancer cells, and cells that exhibit tolerance may withstand this damage by bolstering their DNA repair capabilities. The role of DNA damage repair mechanisms in facilitating radiotherapy resistance has been extensively studied, Radiotherapy works by causing DNA double-strand breaks in cancer cells, and those cells that demonstrate tolerance may endure this damage by enhancing their DNA repair processes (34). Moreover, the role of cytokines in the tumor microenvironment should not be ignored, and they can affect the effect of radiotherapy by modulating the immune response and the inflammatory response (35, 36). As the important regulator of cell cycle regulation and apoptosis, the specific mechanism of the p53 Elevated TGFBI expression is linked to poorer outcomes for patients. Additionally, it also strengthens their resilience against chemotherapy. Focus on understanding the detailed molecular mechanisms behind these pathways, offering hope for the discovery of new therapeutic targets.

TGFBI, transforming growth factor beta-induced, is a protein linked to various types of cancer due to its role in the ECM and its interaction with integrins. This protein may also affect how tumors respond to radiotherapy by altering the tumor microenvironment. TGFBI significantly influences tumor progression and dissemination in NSCLC (37) and is markedly overexpressed in various cancers, notably glioblastoma multiforme (GBM), where it is associated with poorer patient outcomes and increased chemotherapy resistance (38). In esophageal squamous cell carcinoma (ESCC), elevated TGFBI expression correlates with a higher risk of hematogenous relapse and adverse patient outcomes, indicating its potential as a therapeutic target (39). In head and neck squamous cell carcinoma (HNSCC), TGFBI significantly influences cancer stem cell traits and epithelial-mesenchymal transition (EMT), highlighting its role in tumor aggressiveness and metastasis potential (40). These findings align with observations that TGFBI enhances cell proliferation, migration, and invasion in various cancer cell lines, such as lung cancer (41). The construction of the diagnostic nomogram shows that TGFBI has the highest correlation with the prognosis of NSCLC. Therefore, targeting TGFBI could be a promising strategy for overcoming radiotherapy resistance and improving the prognosis of NSCLC patients.

FAS, an essential enzyme involved in the synthesis of fatty acids, has emerged as a significant player in cancer metabolism and the proliferation of tumors. Elevated FAS levels are observed in various cancers, significantly influencing cancer cell metabolism and supporting their growth and survival in nutrient-poor conditions (42). Pharmacological inhibition of FAS shows promise in preclinical models by selectively inducing apoptosis in cancer cells dependent on FAS (43). FAS is also associated with chemotherapy resistance, underscoring its potential in targeting tumor metabolic vulnerabilities (44). Future research should focus on developing selective FAS inhibitors and combining them with current therapies to improve treatment efficacy. As a key regulator, FAS is crucial in controlling apoptosis (45).

Research indicates that PTK6 is often overexpressed in cancers like breast and colon, significantly contributing to cell growth and survival by affecting key signaling pathways (46). PTK6 also influences EMT, a crucial process in cancer metastasis. Thus, targeting PTK6 could disrupt tumor progression and metastasis, suggesting that developing selective small molecule inhibitors or monoclonal antibodies against PTK6 may provide a novel therapeutic approach.

FA2H,while traditionally recognized for its role in lipid metabolism, has recently been implicated in cancer biology, particularly in ovarian cancer. Research indicates that lower FA2H expression is linked to worse outcomes in ovarian cancer, suggesting its role as a tumor growth inhibitor (47). FA2H may affect cisplatin sensitivity by modifying lipid metabolism and cell survival signaling, underscoring the need for further investigation into its dual roles in lipid metabolism and cancer progression. This positions FA2H as a potential biomarker and therapeutic target in ovarian and other cancers.

In our investigation of the transcriptional regulation of key genes, notably, our results revealed that the FAS gene is subjected to the most extensive regulation by various transcription factors. This discovery aligns with previous research highlighting the crucial role of FAS in lipid metabolism and its implications for cancer and metabolic disorders. Furthermore, we identified SP1 as a pivotal transcription factor capable of simultaneously regulating both FAS and PTK6. This dual regulatory role of SP1 underscores its importance in coordinating metabolic and signaling pathways, which may have significant implications in understanding the pathophysiology of diseases where these genes are involved (48, 49). The intricate interplay between these transcription factors and the identification of their target genes highlights the complexity of gene regulation and emphasizes the need for further investigation into the specific roles these genes play in various biological contexts.

Subsequently, we conducted a comprehensive pathway enrichment analysis to identify potential mechanisms underlying the differential prognostic risks observed in chemotherapy patients. GSEA identified significant enrichment of pathways like the Pentose Phosphate Pathway and Proteasome in the low-risk group, while the Phosphatidylinositol signaling system and vascular smooth muscle contraction pathways were enriched in the high-risk group. These findings suggest that metabolic and proteolytic activities may be crucial in the tumor microenvironment of low-risk patients, potentially contributing to better outcomes. In contrast, the activation of pathways related to cell proliferation and vascular function in high-risk patients may indicate a more aggressive disease phenotype, consistent with previous studies linking these pathways to tumor progression and metastasis (50, 51).

The GSVA analysis showed a significant enrichment of pathways KEGG_ALDOSTERONE_REGULATED_SODIUM_REABSORPTION and KEGG_GNRH_SIGNALING_PATHWAY in the high-risk group. This underscores the potential role of hormonal signaling and sodium reabsorption mechanisms in the pathophysiology of NSCLC, which may influence tumor behavior and patient outcomes. The observed increase in mutation load in the low-risk group, alongside the elevated expression of immune checkpoint markers such as CD24, CD28, CTLA-4, and TIGIT in the high-risk group, underscores the complex interplay between genetic mutations and immune escape mechanisms in cancer development. These findings advance our understanding of the molecular mechanisms in NSCLC and propose potential therapeutic targets for high-risk patients, emphasizing the necessity for further research into the clinical implications of these pathways.

Our findings also revealed that the p53 pathway, a well-known tumor suppressor, is involved in the DDR mechanisms, as observed with the small molecule STK899704, which resulted in an increase in the levels of proteins associated with the p53 signaling pathway, subsequently impacting the fundamental DNA damage response mechanisms (52). Furthermore, the engagement of DDR pathways in cisplatin radiosensitization of NSCLC has been demonstrated, where the combination of cisplatin and ionizing radiation led to persistent DNA double-strand breaks (DSBs), suggesting that delayed repair of DSBs contributes to radiosensitization (53).

The regulation of the cell cycle pathway holds a prominent position in NSCLC. For instance, the knockdown of GTSE1, which is involved in cell cycle regulation, enhances radiosensitivity in NSCLC through the DDR pathway by increasing DNA damage post-irradiation (54). Additionally, the involvement of the cell cycle and DDR pathways in leptomeningeal metastasis of NSCLC underscores the complexity and heterogeneity of the disease, suggesting potential therapeutic targets for managing metastatic NSCLC (55).

Moreover, apoptosis pathways are integral to the response of NSCLC to radiotherapy. Defective caspase-3 relocalization in NSCLC cells contributes to resistance against DNA-damage-induced apoptosis, emphasizing the importance of targeting apoptotic pathways to counteract this resistance (56). Jingfukang, a traditional Chinese medicinal formulation, induces apoptosis in circulating tumor cells by activating the ATM/ATR-p53 pathways through reactive oxygen species(ROS), leading to DNA damage. This mechanism offers a promising therapeutic strategy for NSCLC treatment (57).

Overall, understanding the interplay between DDR, cell cycle regulation, and apoptosis pathways provides significant insights on the mechanisms underlying NSCLC’s radiotherapy resistance. These pathways offer promising targets for developing novel therapeutic approaches aimed at augmenting radiotherapy efficacy and improve patient outcomes.

The immune microenvironment is crucial for NSCLC development and treatment response, we performed a comprehensive immune infiltration analysis among patients with distinct prognostic risks to characterize risk-associated immune dynamics, which may hold significant implications for prognostic stratification and therapeutic strategies. Our study identified notable differences in immune cell infiltration between high-risk and low-risk groups, particularly emphasizing naïve B cells and M0 macrophages. These findings align with prior research showing that immune infiltrate composition significantly impacts patient survival rates. For example, Tamminga et al.’s research showed that increased levels of M2 macrophages and active dendritic cells are linked to lower overall OS rates in patients with NSCLC. A higher presence of inactive mast cells and CD4 T-helper cells is associated with better overall survival outcomes (58).This suggests that the undifferentiated macrophages present in the tumor microenvironment may undergo polarization into pro-tumor M2 macrophages. Such a transformation could facilitate tumor progression and contribute to the development of resistance. The distinct infiltration patterns of naïve B cells between risk groups suggest a role in modulating the immune response to NSCLC. This may affect immune response efficacy and patient prognosis.

Understanding the immune landscape of NSCLC provides valuable insights into potential therapeutic targets. Existing studies indicate that the polarization of macrophages, activation of B cells, and changes in the proportions of various B cell subtypes are intricately linked to the regulation of the tumor immune microenvironment and the responses to immunotherapy (59, 60). In the future, targeting the pathways that regulate macrophage polarization or enhancing the activation of naïve B cells could present new strategies for overcoming resistance to radiotherapy. Furthermore, the analysis of immune cell infiltration characteristics can provide strong support for patient stratification management and personalized treatment plans, holding significant potential for translational applications.

It is important to interpret these findings with caution, since experimental validation is essential for establishing their biological significance and potential clinical applications. Results from cell line studies may not fully reflect the mechanisms leading to radiotherapy resistance in actual clinical situations. We recognize that *in vitro* experiments can offer more direct insights into the functional mechanisms of key genes, and we intend to carry out these experiments when circumstances permit. At present, our study is constrained by the availability of clinical samples and experimental resources, leading us to concentrate on bioinformatics analysis utilizing extensive transcriptomic data. We plan to conduct relevant functional experiments in our future research endeavors, aim to validate genes and pathways associated with radiotherapy resistance through cell and animal model experiments.

## Conclusion

In summary, through data analysis, genes related to radiotherapy tolerance in NSCLC were identified. The genes involved in blood coagulation, complement activation, growth factor functionality, and cytokine activity—including interactions between cytokines and their receptors, as well as the chemokine signaling pathway-exhibit significant enrichment. These pathways may contribute to radioresistance, as evidenced by the identification of four crucial genes. A prognostic model based on these genes demonstrated high diagnostic accuracy in predicting patient outcomes, indicating their potential as targets for radiotherapy in NSCLC. Further investigation into these genetic associations could offer significant insights for advancing tumor radiotherapy.

## Data Availability

The datasets presented in this study can be found in online repositories. The names of the repository/repositories and accession number(s) can be found in the article/**Supplementary Material**.

## Glossary

AUC: Area Under the Curve
BP: Biological Process
CC: Cellular Component
DEG: Differentially Expressed Gene
DSBs: Double-strand Breaks
EGFR: Epidermal Growth Factor Receptor
EMT: Epithelial-mesenchymal Transition
ESCC: Esophageal Squamous Cell Carcinoma
FA2H: Fatty Acid 2-Hydroxylation
FAS: Fatty Acid Synthase
GBM: Glioblastoma Multiforme
GEO: Gene Expression Omnibus
GO: Gene Ontology
GSVA: Gene Set Variance Analysis
HNSCC: Head and Neck Squamous Cell Carcinoma
KEGG: Kyoto Encyclopedia of Genes and Genomes
LASSO: Gene Set Variation Analysis
MDA: Mean Decrease Accuracy
MDG: Dean Decrease Gini
MSigDB: Molecular Signatures Database
ncRNA: non-coding RNA
NSCLC: Non-Small Cell Lung Cancer
OS: Overall Survival
PCA: Principal Component Analysis
RBPs: RNA-binding Proteins
ROC: Receiver Operating Characteristic
ROS: Reactive Oxygen Species
ssGSEA: Sample Gene Set Enrichment Analysis
SVM-RFE: Support Vector Machine-Recursive Feature Elimination
TGFBI: Transforming Growth Factor Beta-Induced
TISIDB: Tumor Immune System Interaction Database
MF: Molecular Function
TCGA: The Cancer Genome Atlas Program
