# Supplementary figures and images for: Immunological biomarkers and gene signatures predictive of radiotherapy resistance in non-small cell lung cancer
Source: Front Immunol. 2025 May 29;16:1574113. doi: 10.3389/fimmu.2025.1574113 (PMC12159018; doi:10.3389/fimmu.2025.1574113)

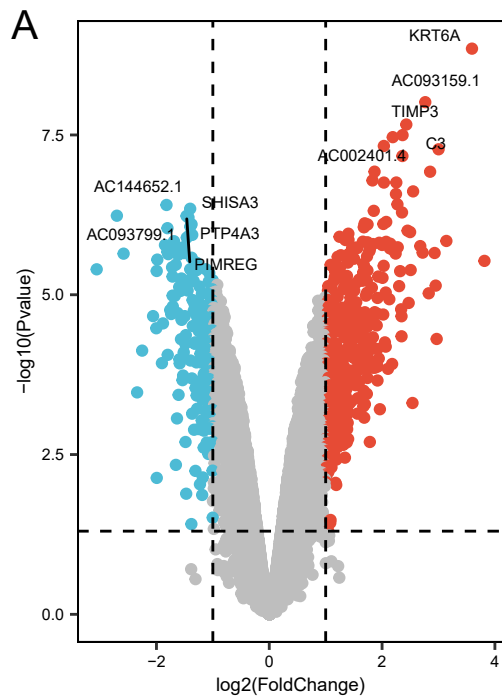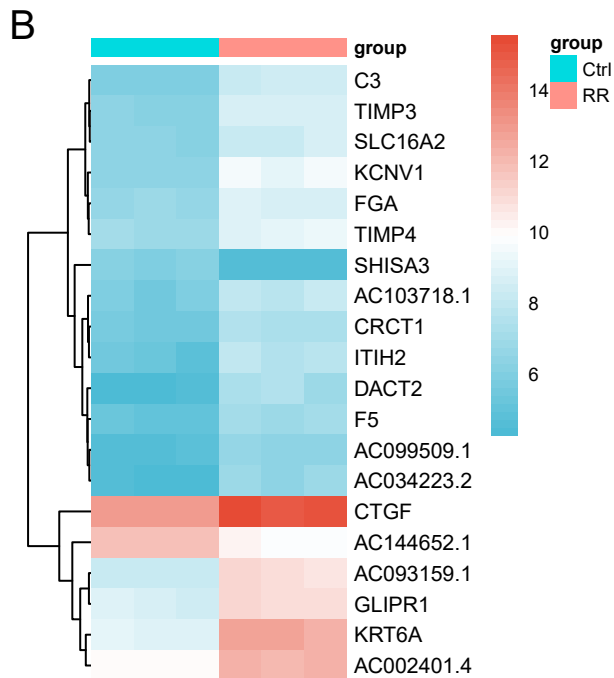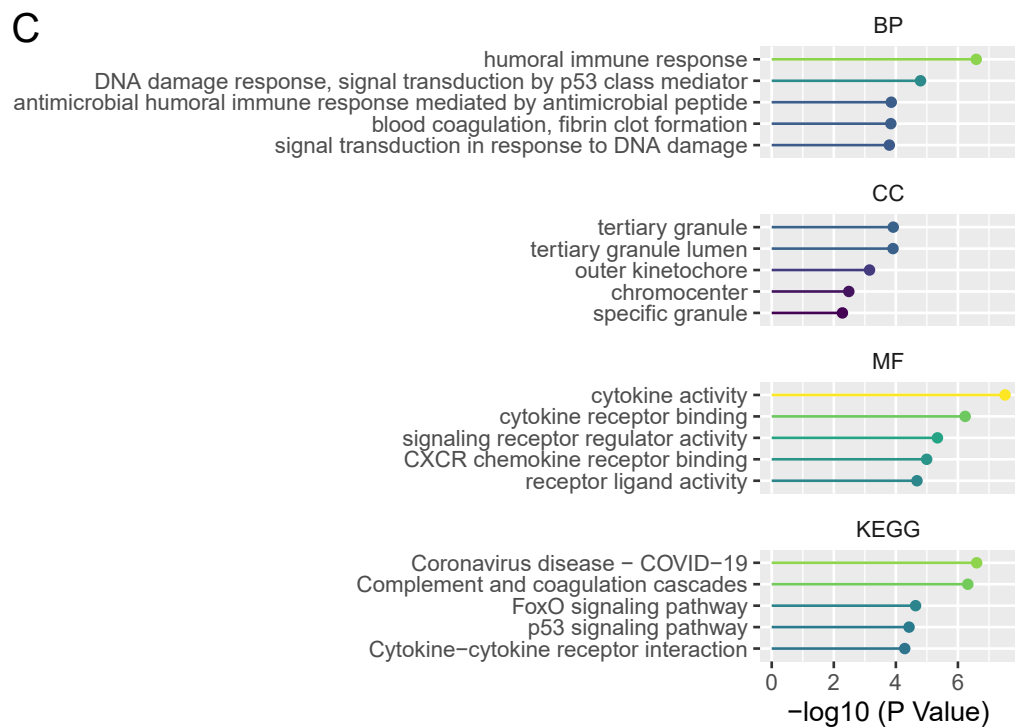

Supplement: Supplementary file 1 [file DataSheet1.zip › Supplementary Material/Supplementary Figures/Supplementary Fig.1.pdf]

A

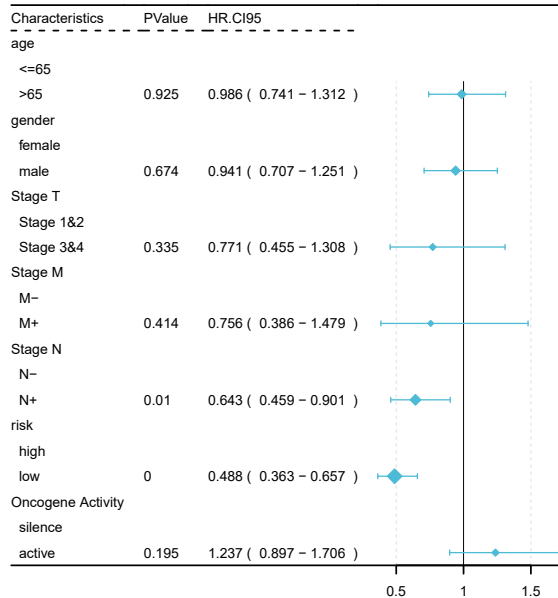

B

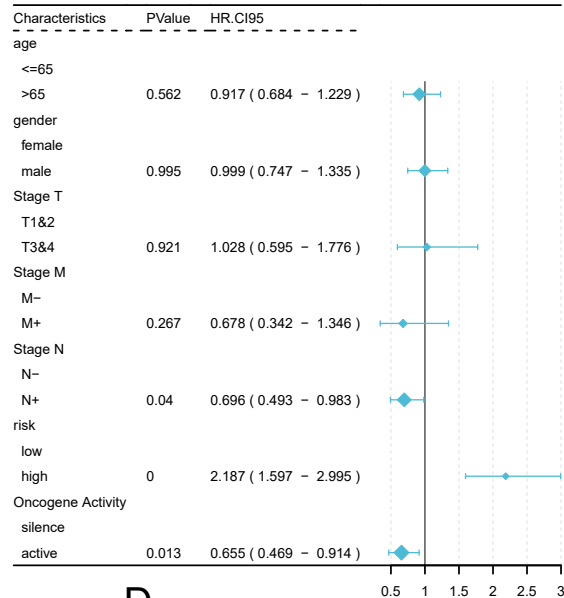

C

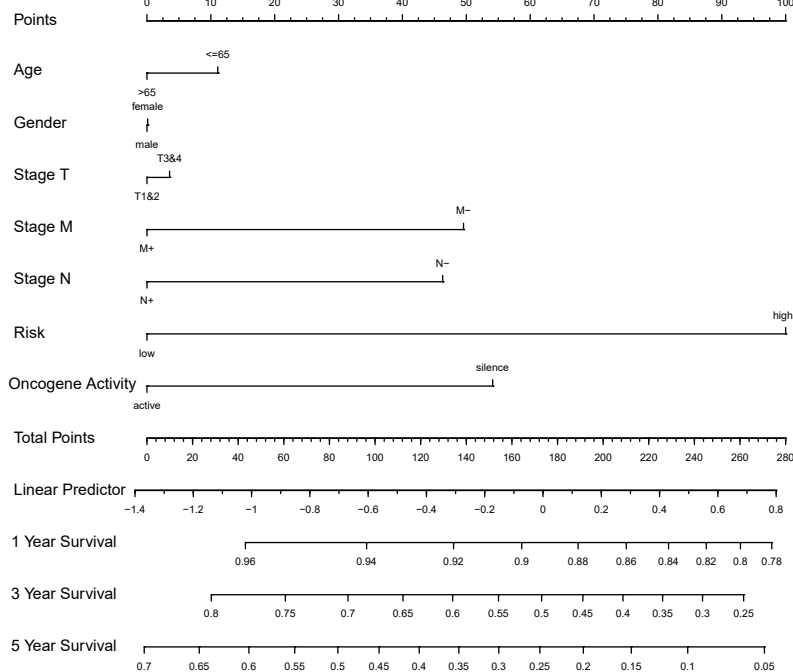

D

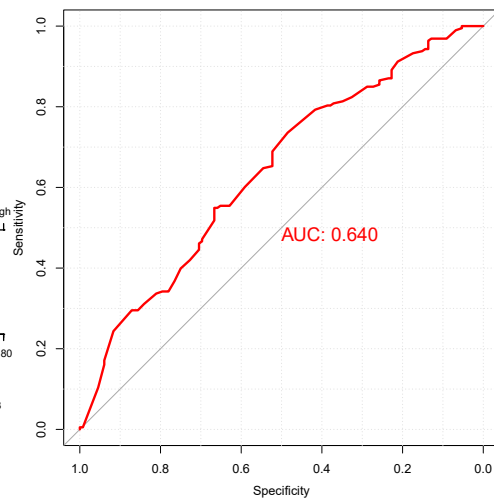

Supplement: Supplementary file 1 [file DataSheet1.zip › Supplementary Material/Supplementary Figures/Supplementary Fig.10.pdf]

A

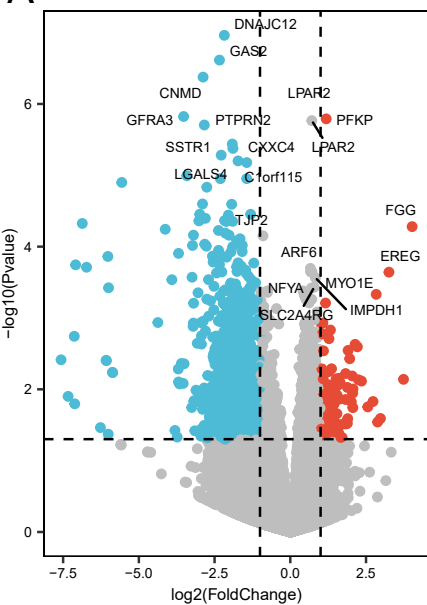

B

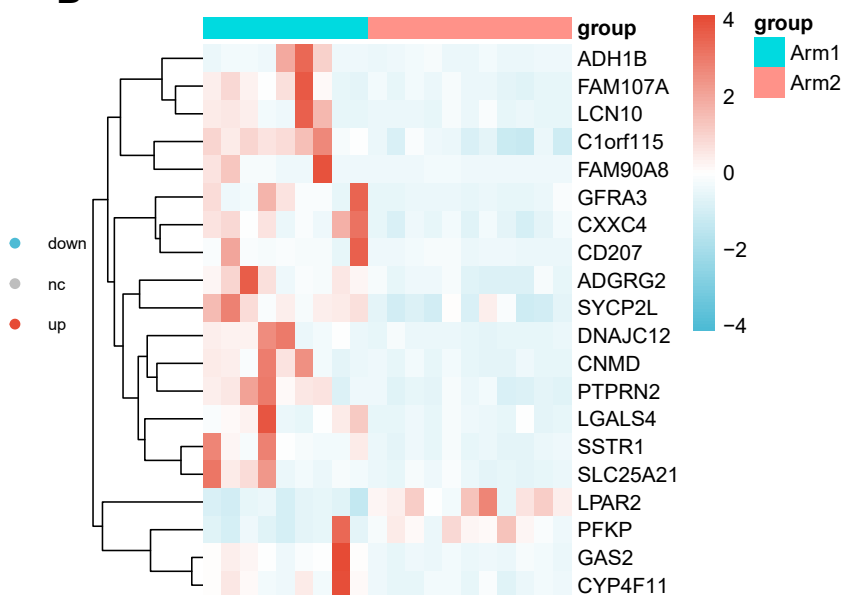

C

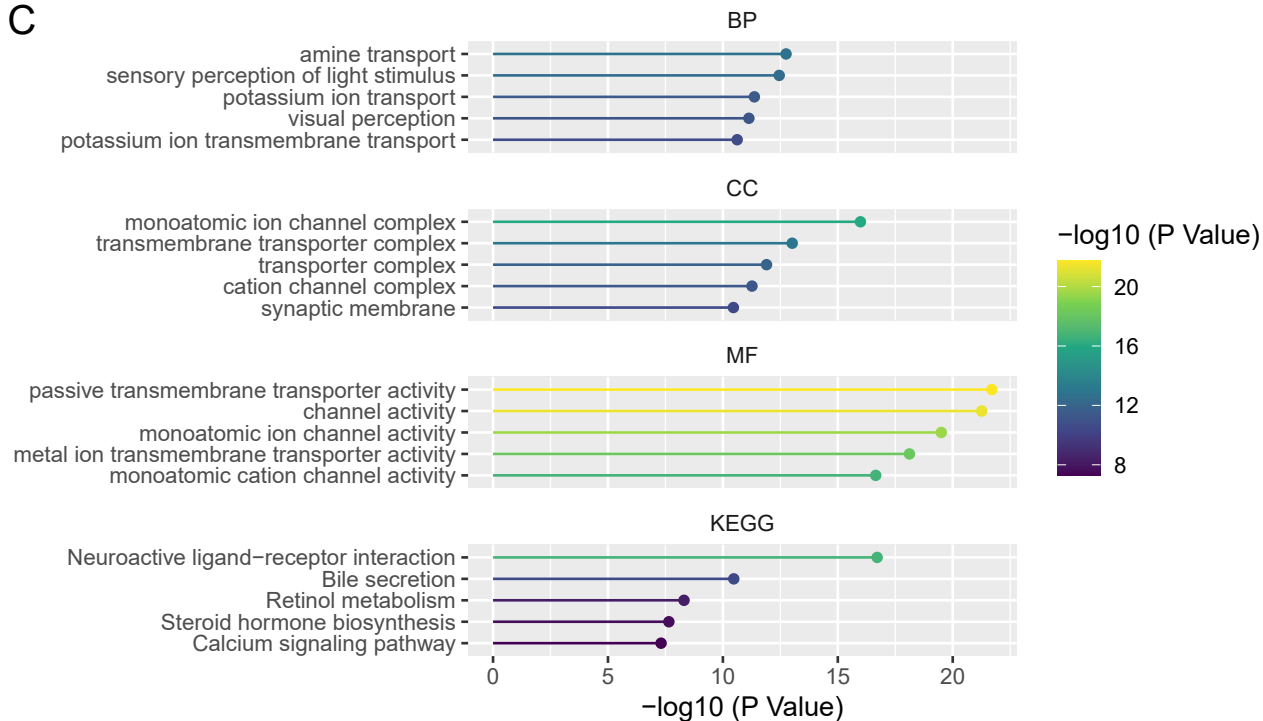

Supplement: Supplementary file 1 [file DataSheet1.zip › Supplementary Material/Supplementary Figures/Supplementary Fig.2.pdf]

**A**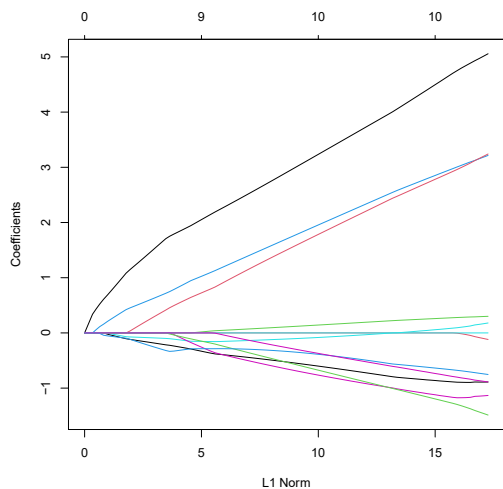**B**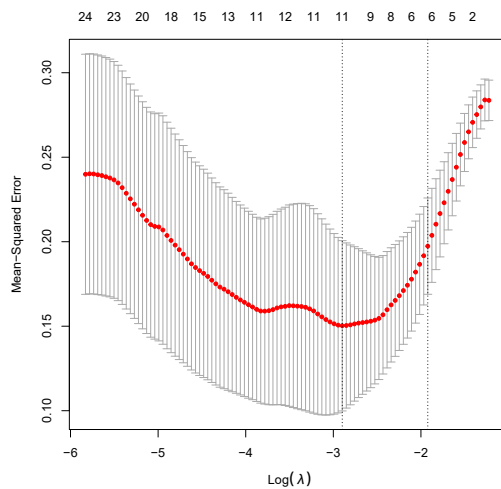**C**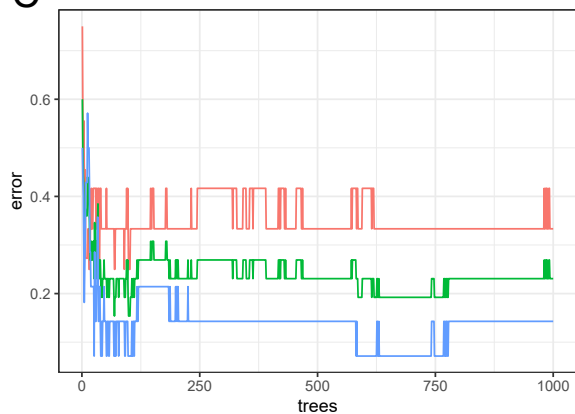**D**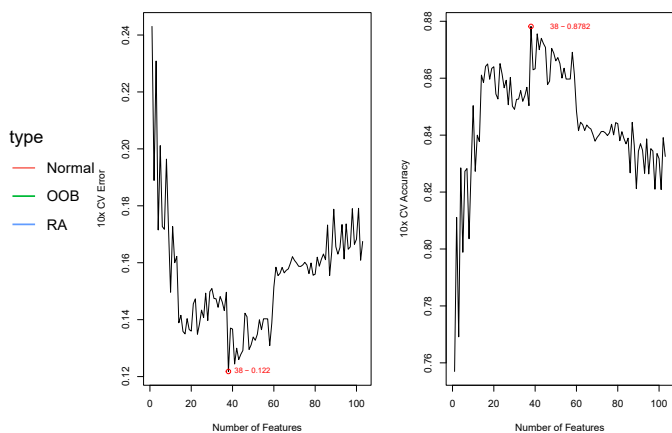**E**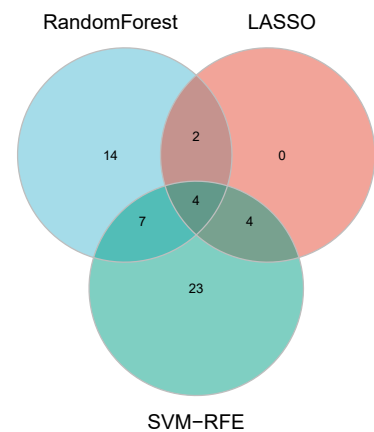**F**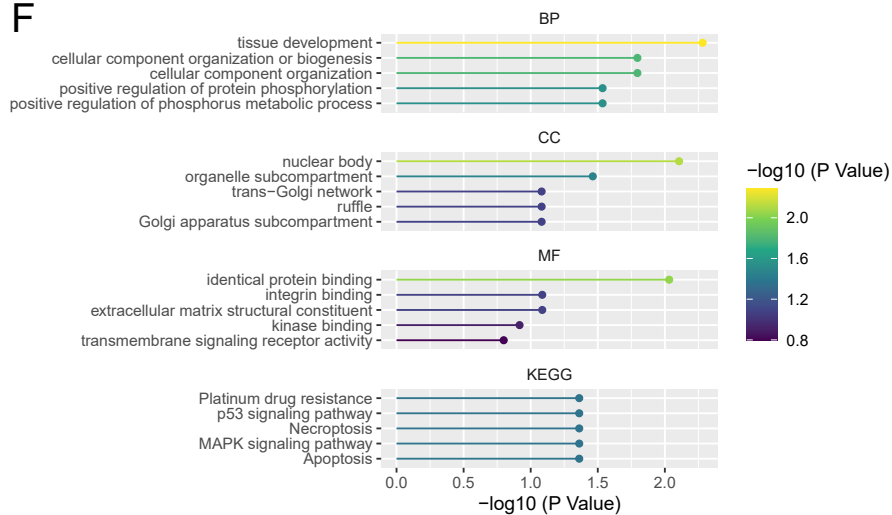

Supplement: Supplementary file 1 [file DataSheet1.zip › Supplementary Material/Supplementary Figures/Supplementary Fig.3.pdf]

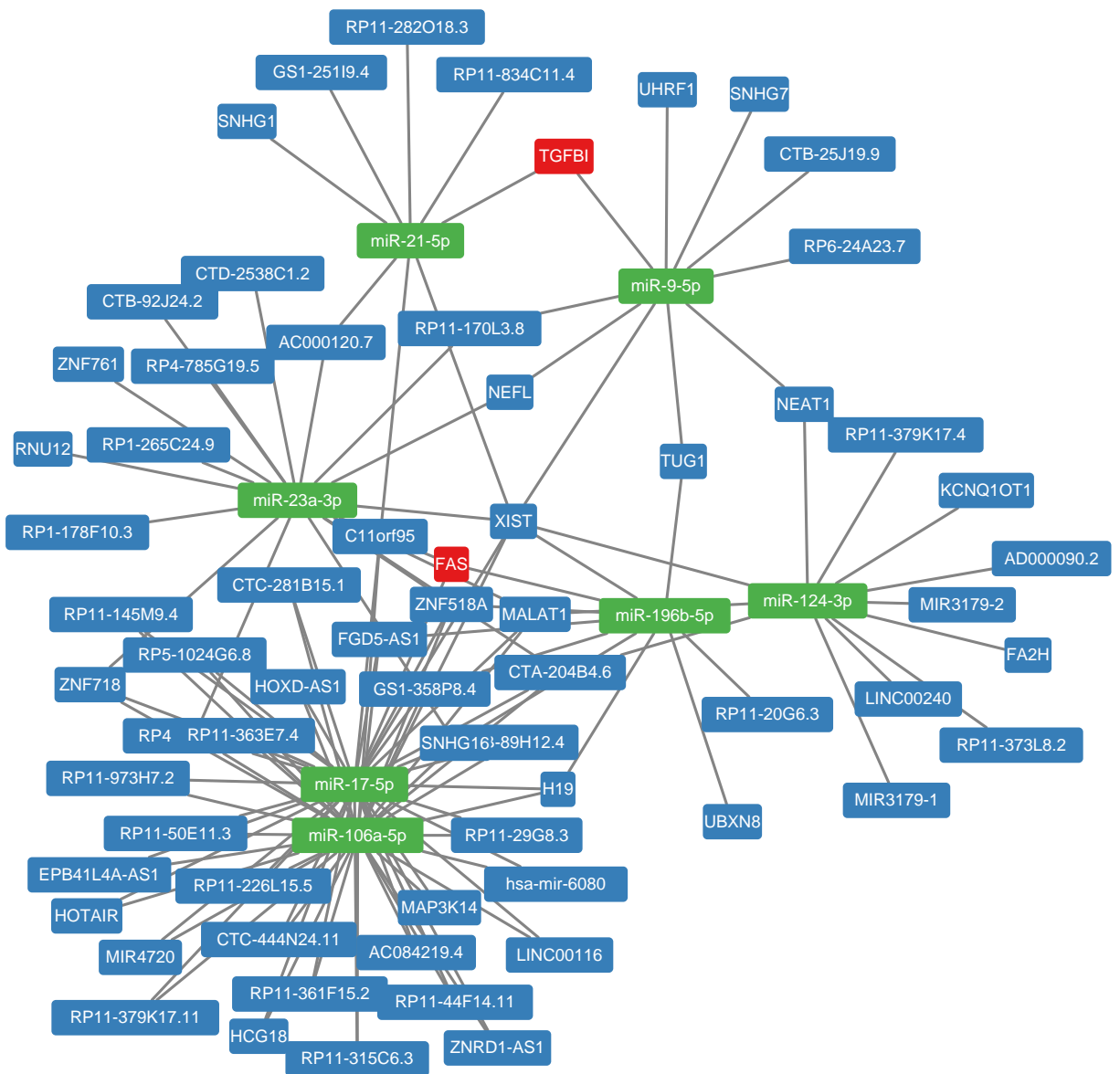

Supplement: Supplementary file 1 [file DataSheet1.zip › Supplementary Material/Supplementary Figures/Supplementary Fig.4 ceRNA.pdf]

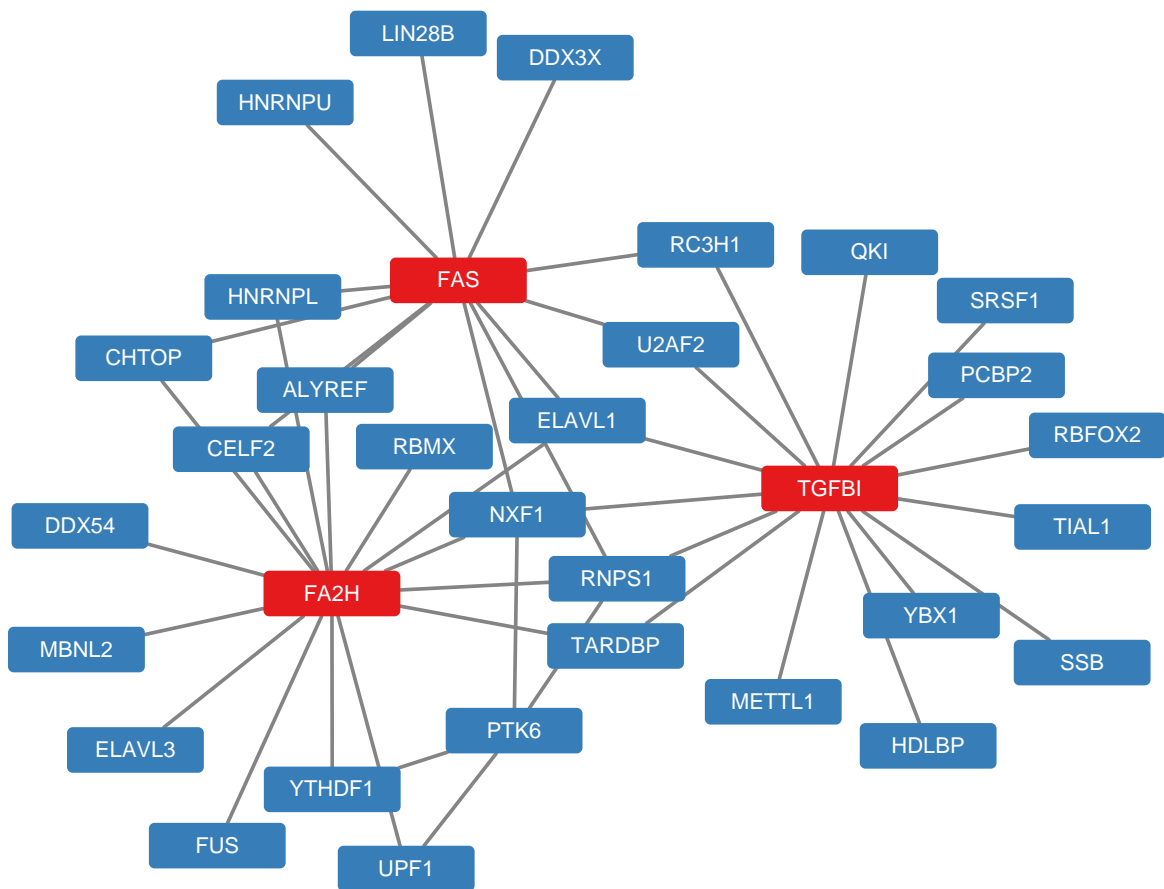

Supplement: Supplementary file 1 [file DataSheet1.zip › Supplementary Material/Supplementary Figures/Supplementary Fig.5A RBP.pdf]

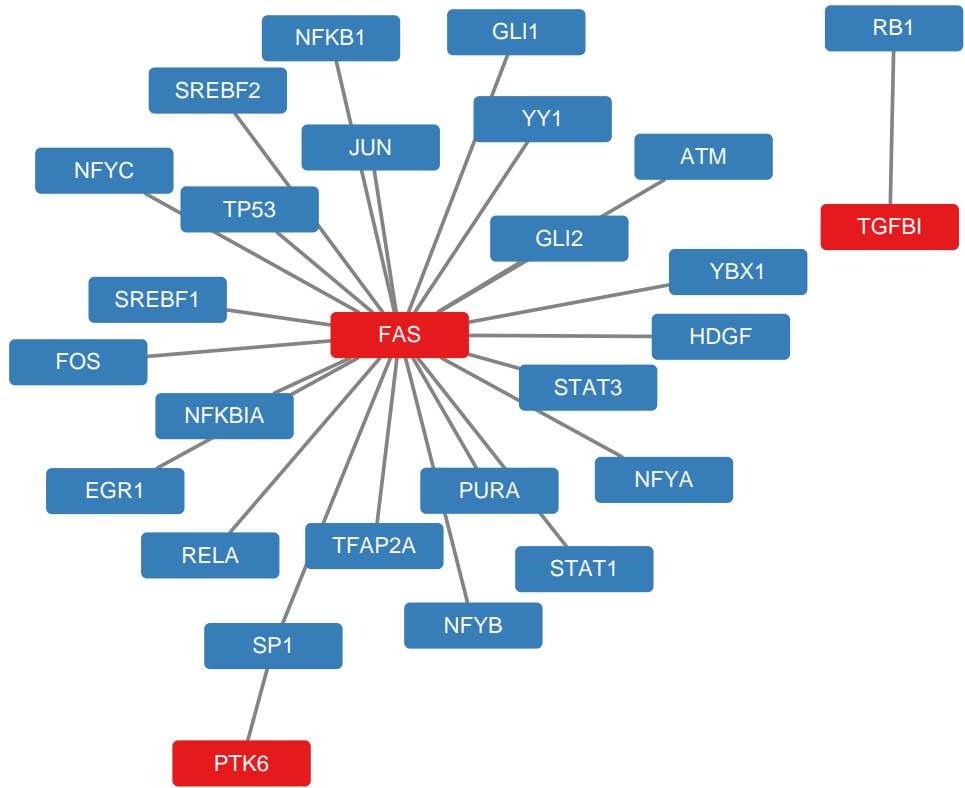

Supplement: Supplementary file 1 [file DataSheet1.zip › Supplementary Material/Supplementary Figures/Supplementary Fig.5B network.pdf]

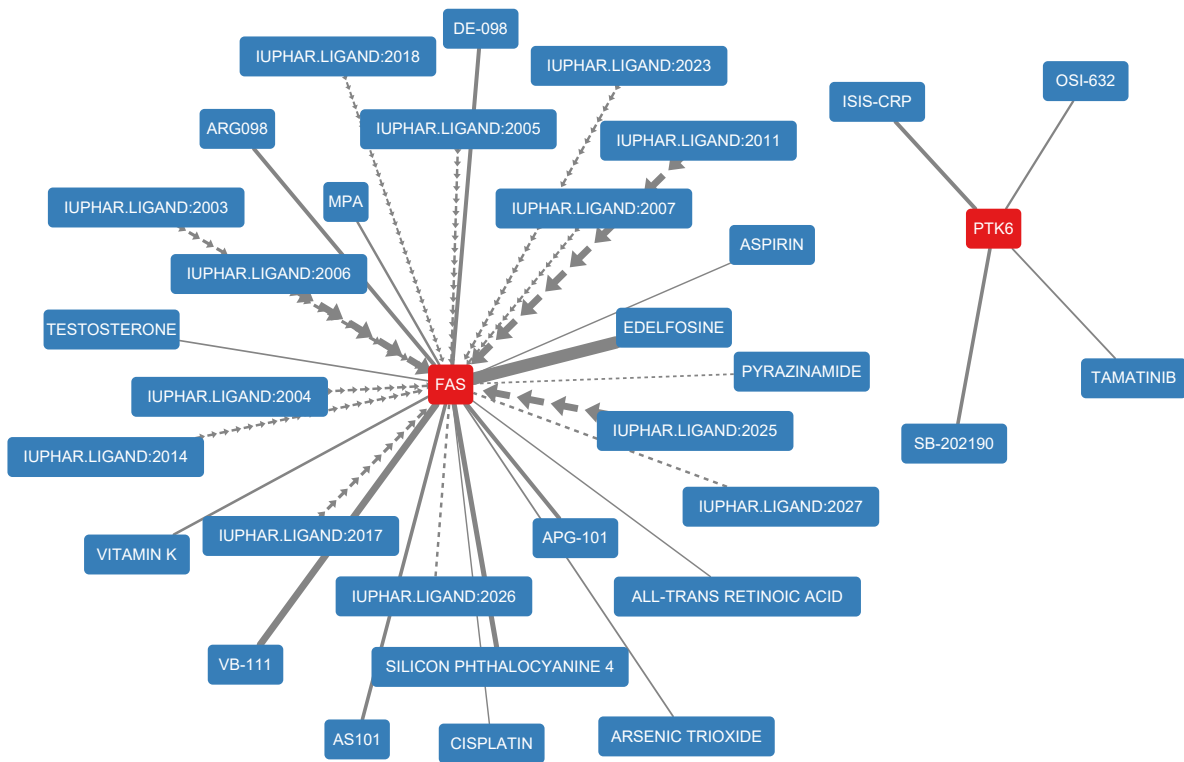

Supplement: Supplementary file 1 [file DataSheet1.zip › Supplementary Material/Supplementary Figures/Supplementary Fig.6.pdf]

A

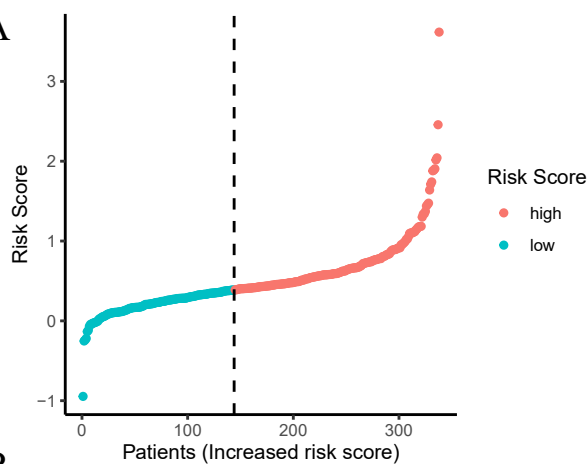

D

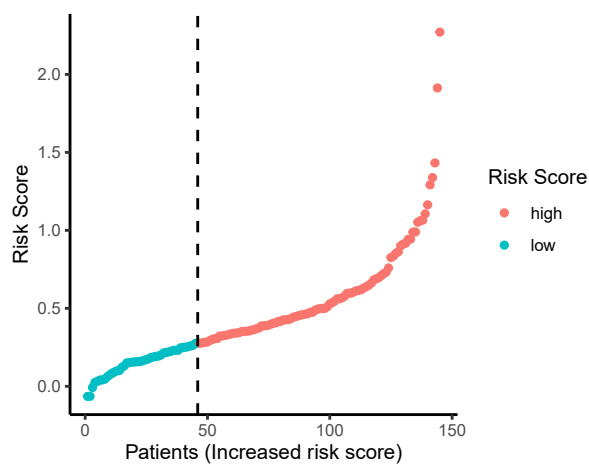

B

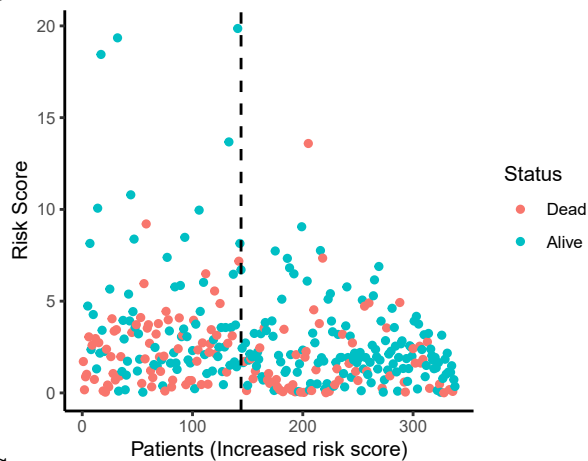

E

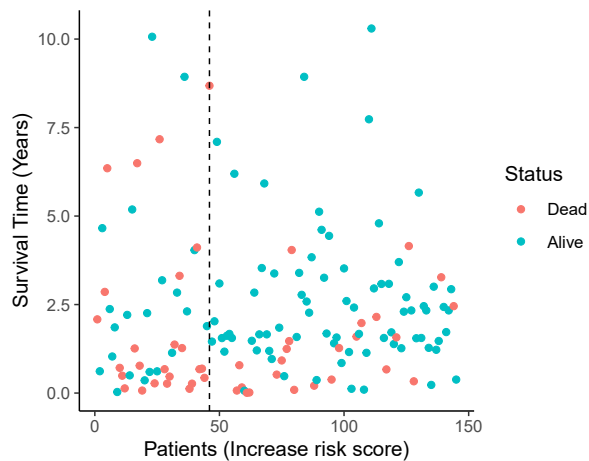

C

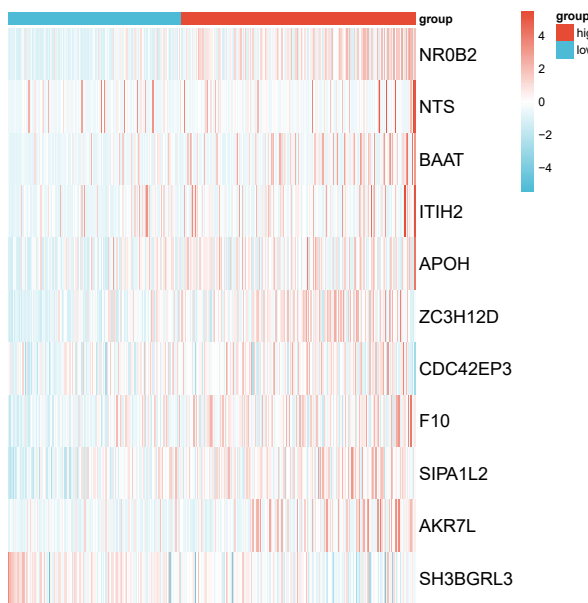

F

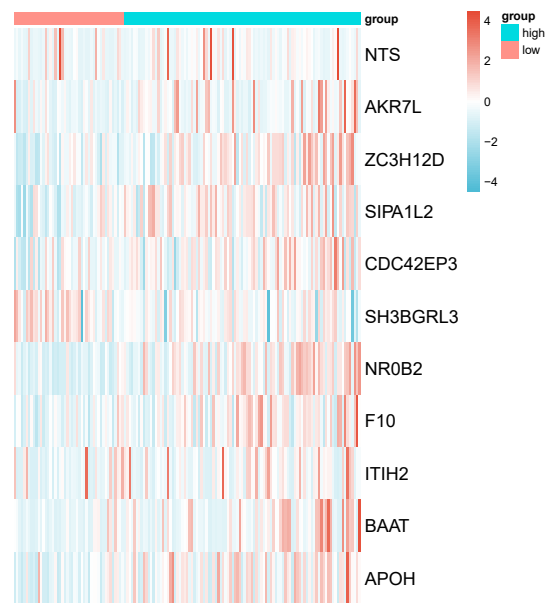

Supplement: Supplementary file 1 [file DataSheet1.zip › Supplementary Material/Supplementary Figures/Supplementary Fig.7.pdf]

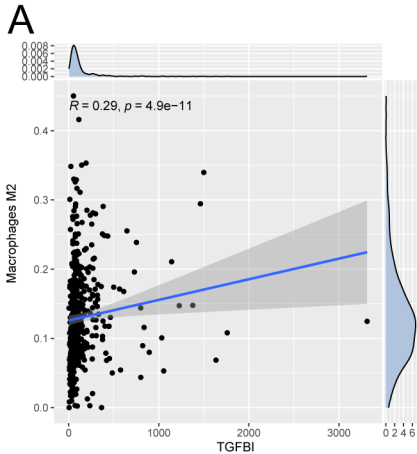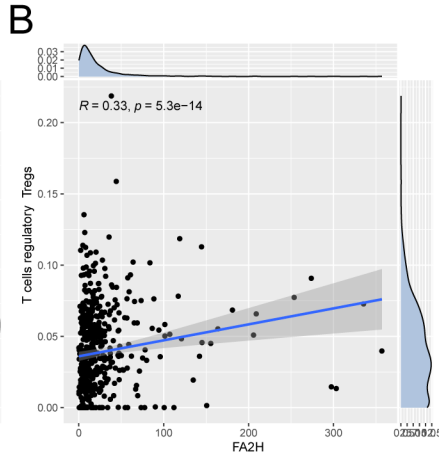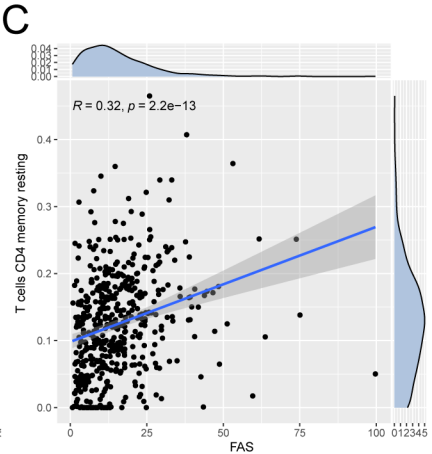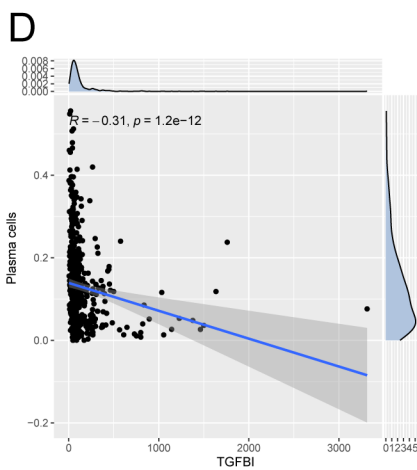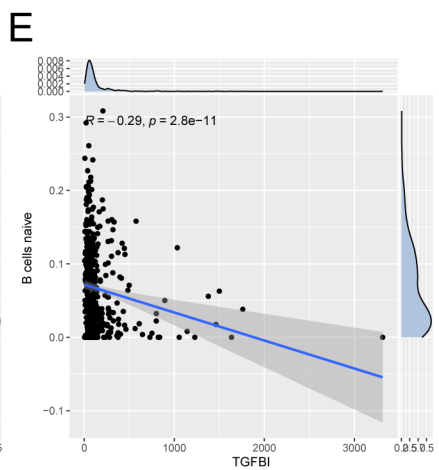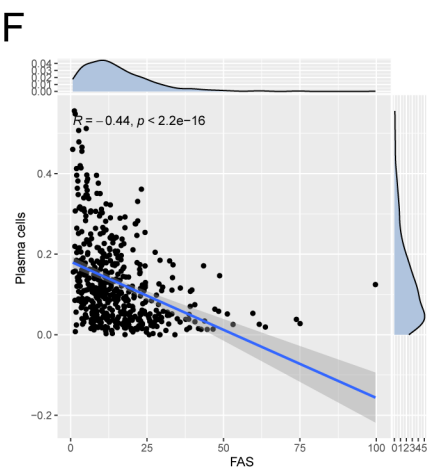

Supplement: Supplementary file 1 [file DataSheet1.zip › Supplementary Material/Supplementary Figures/Supplementary Fig.8.pdf]

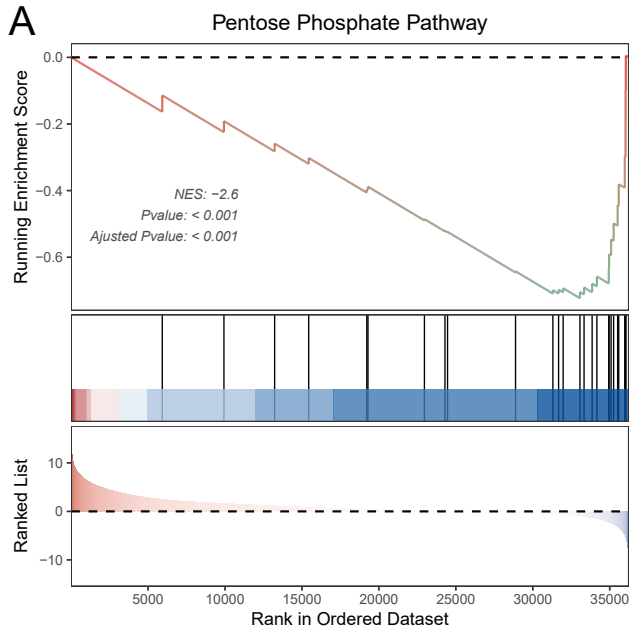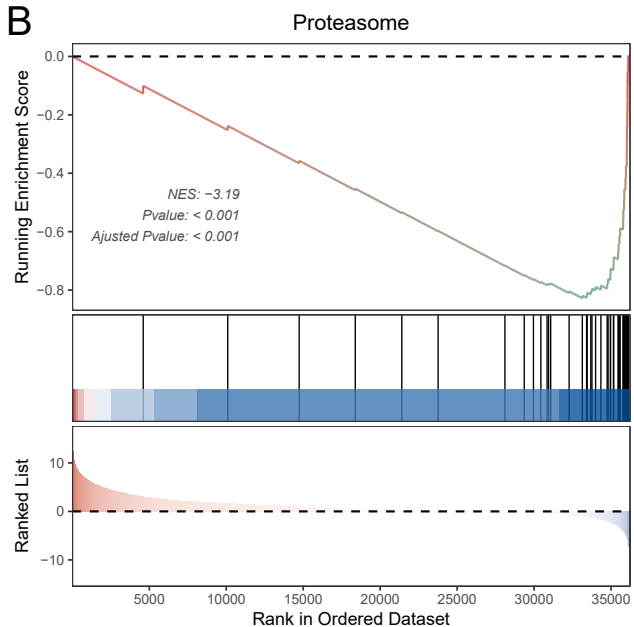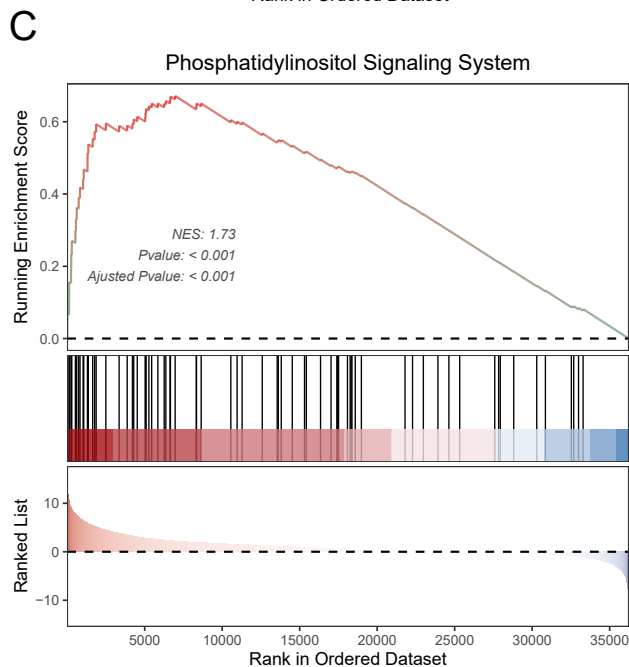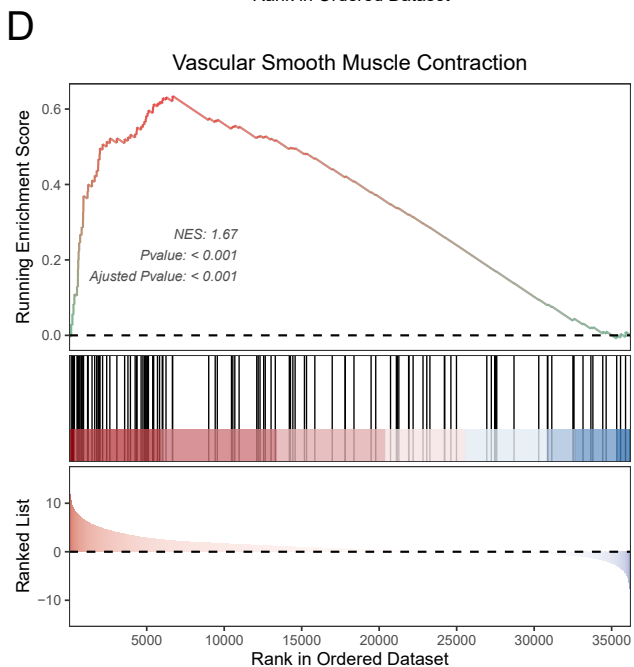

Supplement: Supplementary file 1 [file DataSheet1.zip › Supplementary Material/Supplementary Figures/Supplementary Fig.9.pdf]
